# Expression of human HIPKs in *Drosophila* demonstrates their shared and unique functions in a developmental model

**DOI:** 10.1093/g3journal/jkab350

**Published:** 2021-10-04

**Authors:** Stephen D Kinsey, Justin P Vinluan, Gerald A Shipman, Esther M Verheyen

**Affiliations:** Department of Molecular Biology and Biochemistry, Centre for Cell Biology, Development and Disease, Simon Fraser University, Burnaby, BC V5A 1S6, Canada

**Keywords:** HIPK2, Drosophila, HIPK1, HIPK3, HIPK4, development, imaginal discs, genetic rescue

## Abstract

Homeodomain-interacting protein kinases (HIPKs) are a family of four conserved proteins essential for vertebrate development, as demonstrated by defects in the eye, brain, and skeleton that culminate in embryonic lethality when multiple HIPKs are lost in mice. While HIPKs are essential for development, functional redundancy between the four vertebrate HIPK paralogues has made it difficult to compare their respective functions. Because understanding the unique and shared functions of these essential proteins could directly benefit the fields of biology and medicine, we addressed the gap in knowledge of the four vertebrate HIPK paralogues by studying them in the fruit fly *Drosophila melanogaster*, where reduced genetic redundancy simplifies our functional assessment. The single *hipk* present in the fly allowed us to perform rescue experiments with human *HIPK* genes that provide new insight into their individual functions not easily assessed in vertebrate models. Furthermore, the abundance of genetic tools and established methods for monitoring specific developmental pathways and gross morphological changes in the fly allowed for functional comparisons in endogenous contexts. We first performed rescue experiments to demonstrate the extent to which each of the human HIPKs can functionally replace *Drosophila* Hipk for survival and morphological development. We then showed the ability of each human HIPK to modulate Armadillo/β-catenin levels, JAK/STAT activity, proliferation, growth, and death, each of which have previously been described for Hipks, but never all together in comparable tissue contexts. Finally, we characterized novel developmental phenotypes induced by human HIPKs to gain insight to their unique functions. Together, these experiments provide the first direct comparison of all four vertebrate HIPKs to determine their roles in a developmental context.

## Introduction

Homeodomain-interacting protein kinases (HIPKs) are a family of conserved serine/threonine kinases that are necessary for development in both invertebrate and vertebrate organisms (Blaquiere and Verheyen 2017). In *Drosophila melanogaster*, combined maternal and zygotic loss of the single homolog *hipk* (referred to hereafter as *dhipk*) results in early embryonic lethality, while zygotic loss alone results in pupal lethality (Lee *et al.* 2009a). Experiments performed in mice, which like other vertebrates have four *Hipk* genes (*Hipks1-4*), have demonstrated that knockouts of individual genes are viable, while homozygous loss of both *Hipk1* and *Hipk2* results in embryonic lethality. The viability of single *Hipk* knockouts in vertebrates has been attributed to functional redundancy between the paralogues, where the activity of the remaining HIPKs compensates for the loss (Isono *et al.* 2006). Interestingly, *Hipk1/2* double knockout mice share phenotypes with *dhipk* mutant flies, such as defects in the eye, head, and overall patterning (Isono *et al.* 2006; Lee *et al.* 2009a; Inoue *et al.* 2010).

**Figure 1:**
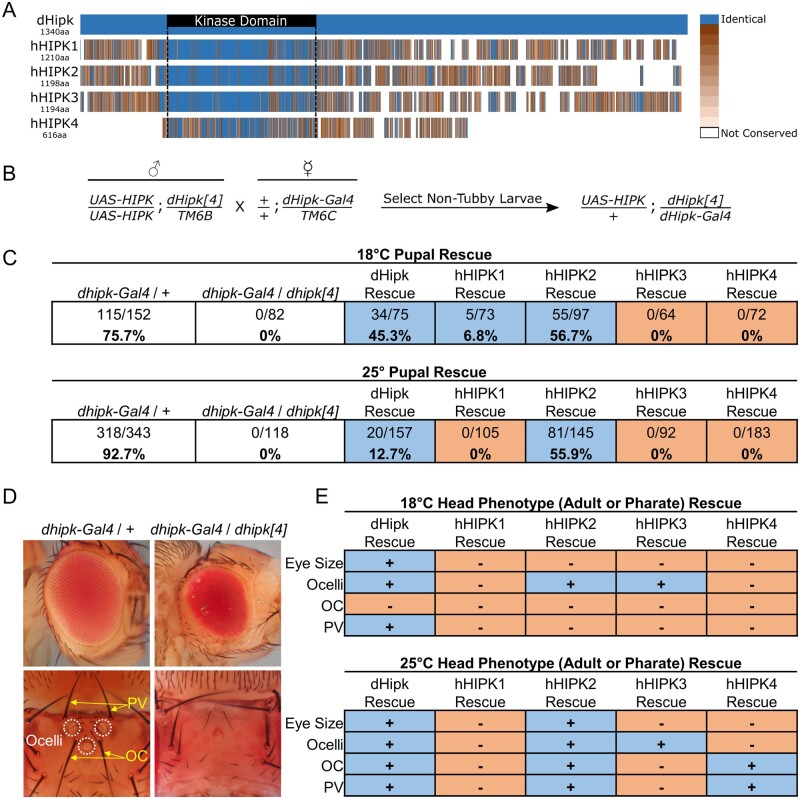
Human HIPKs rescue *dhipk* mutant phenotypes. (A) The amino acid sequence of the four human HIPKs are aligned to dHipk using the NCBI constraint-based multiple alignment tool (COBALT) (Papadopoulos and Agarwala 2007). Dark blue indicates the amino acid at a given position is identical to dHipk at the aligned position, while shades of orange indicate a range between high similarity (dark orange) and low similarity (light orange), and white indicates lack of conservation between the human HIPK and dHipk. (B) The cross scheme used to generate *dhipk* mutant flies that expressed *UAS-hHIPKs* in the *dhipk* expression domain. A male fly homozygous for a *UAS-hHIPK* transgene on the 2nd chromosome and heterozygous for the *dhipk[4]* mutant on the 3rd chromosome over the balancer *TM6B* was crossed to a female fly with a wild-type 2nd chromosome and heterozygous for *dhipk-Gal4* on the 3rd chromosome over the balancer *TM6C*. Both the *TM6C* and *TM6B* balancer chromosomes produce a tubby phenotype, therefore nontubby progeny pupae were scored for each cross. (C) Tables state the number of flies that successfully eclosed from pupal cases at both 18°C and 25°C. White shading indicates the control crosses. Experimental crosses are shown with blue or orange shading to indicate successful or failed eclosion/survival, respectively, with both the ratio and the percent of flies rescued listed. (D) Representative eyes (top) and dorsal head structures (bottom) from heterozygous *dhipk-Gal4/+* flies and transheterozygous *dhipk-Gal4/dhipk[4]* flies, highlighting the reduced eye size, and loss of ocelli, posterior vertical bristles (PV), and ocellar bristles (OC) in *dhipk* mutants. (E) Tables show which Hipks significantly rescue the *dhipk* mutant head phenotypes when expressed in the *dhipk-Gal4/dhipk[4]* mutant background at both 18°C and 25°C, based on graphs and statistical analysis described in Supplementary Figure 2.

The research showing functional redundancy between HIPK1 and HIPK2 provides evidence for their similar developmental roles. It is therefore surprising that comparable studies have not been performed with the other family members. The kinase domain is the region of greatest similarity between vertebrate HIPK paralogs, a similarity that extends to the orthologous dHipk (Figure 1A). In addition, HIPK1, HIPK2, HIPK3, and dHipk share other structural features outside of the kinase domain that have been implicated in protein–protein interactions and in regulating Hipk stability and localization (Rinaldo *et al.* 2008; Blaquiere and Verheyen 2017). Despite the similarity of Hipk proteins, mutant mice demonstrate distinct phenotypes. For example, *Hipk1* knockout mice appear grossly normal, *Hipk2* knockout mice exhibit impaired adipose tissue development, smaller body size, and higher incidence of premature death, *Hipk3* knockout mice exhibit impaired glucose tolerance, and male *Hipk4* knockout mice are infertile due to abnormal spermiogenesis (Kondo *et al.* 2003; Chalazonitis *et al.* 2011; Shojima *et al.* 2012; Sjölund *et al.* 2014; Crapster *et al.* 2020). Unfortunately, these reported phenotypes come from a small number of articles focusing primarily on different tissues, so it is unclear if these variable phenotypes are the result of different spatial temporal expression patterns, different protein functions, or a combination of the two. RNA sequencing projects have demonstrated that human *HIPK1*, *HIPK2*, and *HIPK3* are broadly expressed throughout the adult body and that *HIPK4* is restricted to the brain and testes, however the patterns of *HIPK1-4* expression during development is unclear (Uhlén *et al.* 2015).

The difficulty of uncovering the extent of functional redundancy for vertebrate HIPKs has led to much of the work on these proteins being done in cell culture using exogenously expressed proteins to assess localization, pathway alterations, protein–protein interactions, and altered kinetic activities. While useful for some assays, the cell culture model is unsuitable for comparing developmental functions due to inherent abnormalities in immortalized cell lines, and lack of cellular diversity. One study directly compared all vertebrate HIPKs using cell culture, though its analysis was focused on kinetic activity and cellular localization rather than developmental potential (Van der Laden *et al.* 2015). Despite the lack of direct comparison between vertebrate HIPKs, striking similarities have been observed for the functions of *Drosophila* dHipk and some vertebrate HIPKs, primarily HIPK2, in modulating developmental signaling pathways, including WNT, JNK, Hippo, and JAK/STAT (Rochat-Steiner *et al.* 2000; Hofmann *et al.* 2003, 2005; Lan *et al.* 2007; Lee *et al.* 2009b; Louie *et al.* 2009; Hikasa and Sokol 2011; Huang *et al.* 2011; Swarup and Verheyen 2011; Chen and Verheyen 2012; Poon *et al.* 2012; Shimizu *et al.* 2014).

Recent studies have successfully used the fly as a model to study the functions of human proteins, especially in cases where they fly had reduced redundancy for the candidate gene (McGurk *et al.* 2015; Ugur *et al.* 2016; Link and Bellen 2020; Baldridge *et al.* 2021). We therefore saw the fly as a useful model to compare the functional equivalency of the four wild-type vertebrate HIPKs. The single *Drosophila* dHipk and the abundance of tools available to study developmental signaling in *Drosophila* tissues allow for easy assessment of pathway alterations caused by vertebrate HIPKs. Therefore, we used the fly to determine if the four human HIPKs were capable of performing the same functions in a developmental model. By expressing hHIPKs in both a *dhipk* knockout background, and in multiple tissues of a wild-type genetic background, our comparisons of HIPKs in the fly identified functional similarities between hHIPKs in overall development, as well as unique differences when assessing their activity in identical developing epithelial tissues.

## Materials and methods

### Fly stocks and genetic crosses

Previously described fly strains used in this work are **1:** *w^1118^*, **2:** *dhipk-Gal4* (*hipk[BG00855]*, BDSC #12779), **3:** *UAS-GFP* (BDSC #5431), **4:** *UAS-pc^RNAi^* (BDSC #33964), **5:** *UAS-e(z)^RNAi^* (BDSC #36068), **6:** *UAS-sce^RNAi^* (BDSC #67924), **7:** *UAS-ph-d^RNAi^* (BDSC #63018) **8:** *dhipk^4^* (Lee *et al.* 2009a), **9:** *dpp-Gal4/TM6B* (Staehling-Hampton *et al.* 1994), **10:** *UAS-HA-dhipk^attp40^* (Tettweiler *et al.* 2019), **11:** *eyFLP*; *act>y^+^>Gal4*, *UAS-GFP* (Pagliarini and Xu 2003). The details of how *UAS-myc-hHIPK1^attp40^*, *UAS-myc-hHIPK2^attp40^*, *UAS-myc-hHIPK3^attp40^*, and *UAS-myc-hHIPK4*^attp40^ were generated for this work is detailed in the section titled “Generation of plasmids and transgenic *UAS-hHIPK* fly stocks.” *dhipk* mutant rescue experiments were performed at 18°C and 25°C to determine the ideal Hipk expression levels by modulating the expression of Gal4-driven *UAS-Hipk* constructs, while experiments using *dpp-Gal4* were performed at 29°C to increase *UAS-Hipk* expression. Flies were raised on standard media composed of 0.8 g agar, 2.3 g yeast, 5.7 g cornmeal, and 5.2 mL molasses per 100 ml. “BDSC” is an acronym for the Bloomington Drosophila Stock Center.

### Terminology

As this study investigates human proteins expressed in *Drosophila*, we wanted to clearly indicate which species of protein is specified in each experiment. Throughout this paper, *D. melanogaster* Hipk protein is written “dHipk” while mutants or DNA are referred to as *dhipk*, human HIPKs are written as “hHIPKs,” and in cases where reference is made to proteins from both species, “Hipks” is used.

### Generation of plasmids and transgenic *UAS-hHIPK* fly stocks

Plasmids containing the cDNA for human HIPKs were generously provided by two groups. Dr. Lienhard Schmitz gifted a plasmid containing *hHIPK1* isoform 1, and Dr. Seong-Tae Kim provided us plasmids containing *hHIPK3* isoform 2 and *hHIPK4*. The cDNA for *hHIPK2* isoform 1 was synthesized by GenScript^®^ to match the NCBI reference sequence NM_022740.4. In cases where the gifted cDNAs did not exactly correspond to the translated NCBI reference protein sequences (NP_938009.1 for hHIPK1, NP_001041665.1 for hHIPK3, and NP_653286.2 for hHIPK4), we performed site-directed mutagenesis using the GeneArt™ Site-Directed Mutagenesis PLUS system to correct the cDNA sequence. The cDNAs that corresponded to these reference sequences were then tagged with N-terminal Myc-epitope tags before being cloned into a pUAST-attB backbone vector using NotI and XhoI restriction sites for *hHIPK1* and *hHIPK2*, BglII and KpnI sites for *hHIPK3*, and BglII and XhoI sites for *hHIPK4.* The four pUAST-attB-Myc-hHIPK plasmids were then sent to BestGene Inc. for injection into *Drosophila* embryos containing an attP40 site, allowing for stable integration to identical sites on the second chromosome. The resulting fly stocks each contain a single *Myc-hHIPK* cDNA under the control of a UAS promoter that is expressed in any cell expressing a Gal4 transcription factor.

### Adult *Drosophila* imaging and scoring rescue phenotypes

The pharate pupae and viable adults from the *dhipk* mutant viability rescue experiment were collected, and if necessary, gently removed from their pupal cases with dissecting tweezers before being immediately placed in 70% ethanol and stored at −20°C for preservation until they were photographed for the assessment and quantification of head phenotypes. Six randomly selected female flies from each cross were used for phenotype quantification. To image these flies, we used an 8-well BD Falcon CultureSlide (REF 354118) modified to have each well filled 1/3 with SYLGARD™ 184 (Supplementary Figure S5). Insect pins were bent at 90° and pinned into the solidified SYLGARD so that the 90° bend was located near the top of the plastic well. Immediately before imaging, flies were removed from 70% ethanol at −20°C to individual wells filled with 70% ethanol at room temperature and pinned to the planted insect pins while remaining submerged. The slides were then topped off with excess 70% ethanol before a coverslip was placed atop the wells. A resulting slide contained six female flies of the same genotype pinned at a stable position for imaging near the surface of the coverslip, while remaining submerged in ethanol. The ethanol was required to prevent flies drying out during imaging, and the coverslip was required to prevent vibrations on the surface of the ethanol that interfered with imaging. The same six flies were photographed three times to capture each eye (two images per fly) and the top of the head (one image per fly). Lighting was provided by an LED strip modified to encircle the CultureSlide, and a folded white tissue was placed under the CultureSlide to obtain a white/grey background.

Adult wings and legs were dissected in ethanol, then gently dried on a paper towel before being submerged in a small drop of Aquatex^®^ (Sigma-Aldrich #1.08562) and covered in a coverslip. Small weights (EM stubs) were then placed on the coverslips while being heated to 60°C for 1 h. All adult phenotypes were imaged using a Zeiss Axioplan2 microscope with an Optika C-P6 camera system.

To determine pupal lethality in the *dhipk* mutant rescue experiment, crosses were performed with 24-h egg lays, and all non-Tubby pupal cases were scored as eclosed or pharate 5 days after flies were expected to have eclosed.

### HIPK protein sequence alignment

After confirming that our cDNA sequences correctly translated to the NCBI reference protein sequences for hHIPK1 isoform 1 (NP_938009.1), hHIPK2 isoform 1 (NP_073577.3), hHIPK3 isoform 2 (NP_001041665.1), hHIPK4 (NP_653286.2), and dHipk isoform A (NP_612038.2), each of the hHIPK sequences were individually compared to dHipk using the NCBI COBALT tool (Papadopoulos and Agarwala 2007). dHipk was set as the anchor. The FASTA alignment for this comparison was then downloaded and opened in Jalview (version 2.11.1.2) to extract the numerical conservation data between each of the hHIPKs and dHipk individually (Waterhouse *et al.* 2009). The numerical conservation data (from 0 = no conservation, to 11 = identical amino acid) was then extracted and sent to Microsoft Excel (Excel 365), where numerical columns were converted to a color gradient. An image of the alignment was then exported as a PNG to Inkscape (version 0.92.4) for annotation, based on the NCBI annotation of the kinase domain.

### Immunocytochemistry and microscopy

Late third instar larval imaginal discs were dissected and stained using previously described methods (Blaquiere *et al.* 2018). The following primary antibodies were used: mouse anti-Ubx (1:50, DSHB Ubx FP3.38) mouse anti-Scr (1:50, DSHB anti-Scr 6H4.1), mouse anti-Arm (1:10 DSHB N27A1 Armadillo), mouse anti-Wg (1:50, DSHB 4D4), rabbit anti-PH3 Ser10 (1:500, Cell Signaling #9701S). Imaginal discs were imaged on a Zeiss LSM 880. Images were processed in FIJI.

### Clonal analysis

FLP-out clones expressing UAS-Hipks positively marked with RFP were generated by exposing 1st instar larvae of the genotype *hsflp^112^/+; 10xStat92E-GFP/UAS-Hipk; actin>CD2>Gal4, UAS-RFP/+* to a 37°C water bath for 12 min, followed by incubation at 29°C until larvae reached the wandering 3rd instar stage, as performed by Wong *et al.* (2019). Wing imaginal discs were then dissected, stained, and imaged as above.

### PH3 and TUNEL assay quantification using wing imaginal discs

Dual PH3 and TUNEL assay staining was performed by first completing the normal wing disc dissection, fixing, washing, and primary antibody treatment protocol noted previously for PH3 (1:500 in block, Cell Signaling #9701S). Before secondary antibody staining, TUNEL staining was performed using the Roche *In Situ* Cell Death Detection Kit, TMR Red (Version 12, Cat. No. 12 156 792 910). Once the tissues were washed after the primary antibody treatment, the wash was removed, and 100 µl of combined TUNEL assay components (92.7 µl labeling solution + 8.3 µl enzyme solution) was added to the tissues in a 1.6 mL Eppendorf tube, along with 1:1000 goat α-rabbit fluorophore conjugated secondary antibody (Jackson ImmunoResearch, product # 711-605-152). The tissues were then incubated overnight (∼16 h) on a rocker in the dark at 4°C. Staining regents were then removed, and samples were rinsed quickly with PBT before staining for 30 min with 1:500 DAPI solution. After DAPI staining, four more 10-minute washes were performed before wing discs were separated from other tissues and mounted in 70% glycerol on microscope slides. Wing imaginal discs were imaged as described in the previous section. Using FIJI (Schindelin *et al.* 2012, 2015; Schneider *et al.* 2012), the area of the whole wing imaginal disc and *dpp-GFP* domains were measured, and PH3 or TUNEL positive cells were counted within each region automatically using the Analyze → Analyze Particles tool after thresholding. The change in concentration of PH3 or TUNEL positive cells between the dpp-GFP domain and the rest of the disc was then calculated.

### RNA extraction and qPCR

RNA extractions were performed using the Qiagen RNeasy^®^ Plus Mini Kit (#74134). RNA that was used to confirm reduced *dHipk* mRNA in *dhipk* mutant and rescue crosses, as well as verify the correct *hHIPK* expression in the rescue crosses, was collected from four combined wandering 3rd instar larvae (two male and two female) for each cross. Larvae were washed in PBS before being spot dried on a clean paper towel and transferred to 300 µl buffer RLT Plus, supplemented with freshly added β-mercaptoethanol to 1%. Larvae were homogenized with pestles by hand in 1.6 mL tubes before being centrifuged for 3 min at maximum speed to pellet debris. Supernatant was transferred to a gDNA Eliminator spin column, with the remaining RNA extraction steps following the manufacturer’s instructions.

cDNA synthesis was performed using ABM^®^ OneScript^®^ Plus cDNA Synthesis Kit (#G236). For each sample, 100 ng mRNA was used in combination with Oligo (dT) primers to perform first-strand cDNA synthesis of poly-adenylated mRNA following manufacturer’s instructions. Resulting cDNA was diluted 1:5 before being used for qPCR.

qPCR for each sample/primer mix was performed in triplicate with 10 µl samples (technical replicates), utilizing Bioline’s sensiFAST SYBR Lo-ROX Kit (#BIO-94005) on an Applied Biosystems QuantStudio 3. One microliter of diluted cDNA was used per reaction. Primers targeting *rp49* were used as reference targets.

### Primers

*rp49* F: AGCATACAGGCCCAAGATCG

*rp49* R: TGTTGTCGATACCCTTGGGC

*dhipk* F: GCACCACAACTGCAACTACG

*dhipk* R: ACGTGATGATGGTGCGAACTC

*hHIPK1* F: GACCAGTGCAGCACAACCAC

*hHIPK1* R: GCCATGCTGGAAGGTGTAGG

*hHIPK2* F: GTCCACCAACCTGACCATGA

*hHIPK2* R: GGAGACTTCGGGATTGGCTA

*hHIPK3* F: GACATCAGCATTCCAGCAGC

*hHIPK3* R: GCTGTCTTCTGTGCCCAAAG

*hHIPK4* F: GCCTGAGAACATCATGCTGG

*hHIPK4* R: GCGACTGGATGTATGGCTCC

## Results

### hHIPK1 and hHIPK2 rescue *dhipk* mutant lethality

As a first step in characterizing hHIPK functions in *Drosophila*, we wanted to test whether expression of *hHIPKs* using the Gal4/UAS system could rescue phenotypes caused by loss of *dhipk*. To do this, we combined two *dhipk* mutant alleles, *dhipk[4] and dhipk-Gal4*, to generate a transheterozygous (heteroallelic) knockout of *dhipk* (Figure 1B). The *dhipk[4]* mutant has a deletion removing 9 out of the possible 10 exons (Lee *et al.* 2009a), while the *dhipk-Gal4* mutant generated by the Drosophila Gene Disruption Project (insertion #BG00855) contains a Gal4 coding sequence inserted upstream of *dhipk* that effectively prevents its expression when combined with the *dhipk[4]* allele (Supplementary Figure S1, A–D) (Bellen *et al.* 2004, 2011). In subsequent sections, *dhipk[4]*/*dhipk-Gal4* mutant flies are simply referred to as “*dhipk* mutants.” These *dhipk* mutants are 100% lethal prior to pupal eclosion, with pharate pupae dissected from pupal cases showing reduced eye size, loss of ocelli, and missing ocellar bristles (Figure 1, C and D). This knockout approach has two main benefits. First, it disrupts endogenous *dhipk* expression while allowing expression of *UAS*-driven transgenes in the endogenous *dhipk* domain due to the insertion of Gal4 coding sequences in the *dhipk* locus (Supplementary Figure S1, C and D). Second, this approach reduces the effect of secondary mutations present on chromosomes carrying the individual *dhipk* mutant alleles that may contribute to lethality when made homozygous.

To confirm that the *dhipk-Gal4* allele was capable of driving UAS-transgene expression in the appropriate tissues and stages, we first expressed a wildtype UAS-*dhipk* cDNA construct in the *dhipk* mutant background (Figure 1C). We expected a phenotypic rescue if the *dhipk-Gal4* allele correctly drove UAS expression in the endogenous *dhipk* domains. We raised these crosses at both 18°C and 25°C to assay the effects of two levels of transgene expression, since the activity of Gal4 and therefore level of expression of UAS transgenes is enhanced at higher temperatures (Duffy 2002). This was essential to determining optimal conditions, since our previous work has shown that overexpression of dHipk in a wildtype background at 29°C causes numerous phenotypes, including tumorigenic effects (Blaquiere *et al.* 2018; Wong *et al.* 2019, 2020). As expected, the majority of control flies heterozygous for the *dhipk-Gal4/+* allele successfully eclosed from pupae (92.7% at 25°C and 75.7% at 18°C) and 0% of *dhipk* mutant flies eclosed at either temperature, with death occurring at or before the pupal stage (Figure 1C). In the *UAS-dhipk* rescue experiment, 12.7% of flies eclosed at 25°C, and 45.3% of flies eclosed at 18°C, indicating that the *dhipk-Gal4* allele drives *UAS-dhipk* in a spatial and temporal pattern sufficiently similar to endogenous *dhipk* expression.

We next tested the ability of the four *UAS-hHIPK* transgenes to rescue *dhipk* mutant lethality (Figure 1C). To maintain consistency of transgene expression, we utilized targeted integration to insert each human and fly *HIPK* cDNA into the genome on the second chromosome at the engineered attp40 landing site as described in the methods. We found that *UAS-hHIPK1* rescued 6.8% of *dhipk* mutants at 18°C, while it was unable to rescue at 25°C. In contrast, *UAS-hHIPK2* rescued the lethality of 56.7% of *dhipk* mutants at 18°C, and 55.9% at 25°C, which was more effective than the rescue by *UAS-dhipk*. Finally, neither *UAS-hHIPK3* nor *UAS-hHIPK4* rescued the lethality of *dhipk* mutants (Figure 1C).

### *hHIPKs* variably rescue *dhipk* mutant patterning phenotypes

Only *UAS-hHIPK1* and *UAS-hHIPK2* were able to rescue *dhipk* mutant lethality, however it was possible that the other hHIPKs could rescue minor *dhipk* mutant patterning phenotypes in fully formed, yet inviable, pharate adults dissected from their pupal cases. *dhipk* mutant flies that develop to the pharate adult stage have reduced compound eye size, and are missing the three ‘simple eyes’ called ocelli on the top of their heads (Figure 1D) (Lee *et al.* 2009a; Blaquiere *et al.* 2014). Ocellar and posterior vertical bristles are also lost in *dhipk* mutant pharate adults. Combined, the eye, ocelli, and bristle phenotypes are the most obvious external changes on pharate *dhipk* mutant flies. Therefore, we asked if *UAS-hHIPKs* could rescue these phenotypes. As with the *dhipk* mutant lethality rescue experiments, we carried out these crosses at both 18°C and 25°C to modulate the degree of Gal4-driven expression of the transgenes (Figure 1E, Supplementary Figure S2).

While the rescue of *dhipk* mutant lethality by *UAS-dhipk and UAS-hHIPKs* was more effective at 18°C than it was at 25°C, this was not true for the head phenotypes. *UAS-dhipk* was able to significantly rescue each *dhipk* mutant phenotype when raised at 25°C but failed to rescue the ocellar bristle loss at 18°C (Figure 1E, Supplementary Figure S2). For the human HIPKs, *UAS-hHIPK1* was unable to rescue any head phenotype at either 18°C or 25°C, despite rescuing lethality at 18°C. *UAS-hHIPK2* significantly rescued all phenotypes at 25°C, but only rescued the loss of ocelli at 18°C. Finally, while *UAS-hHIPK3 and UAS-hHIPK4* were unable to rescue *dhipk* mutant lethality, *UAS-hHIPK3* rescued the loss of ocelli at both temperatures, and *UAS-hHIPK4* rescued the loss of ocellar bristles and posterior vertical bristles at 25°C only. In addition, *UAS-hHIPK4* caused a significant reduction in eye size compared to the *dhipk* mutant phenotype alone at both temperatures (Supplementary Figure S2). Together, these rescue experiments show that only human HIPKs 1 and 2 are capable of rescuing *dhipk* mutant lethality, while each of the human HIPKs can rescue a subset of the *dhipk* mutant head phenotypes.

### hHIPKs act on dHipk target pathways

**Figure 2:**
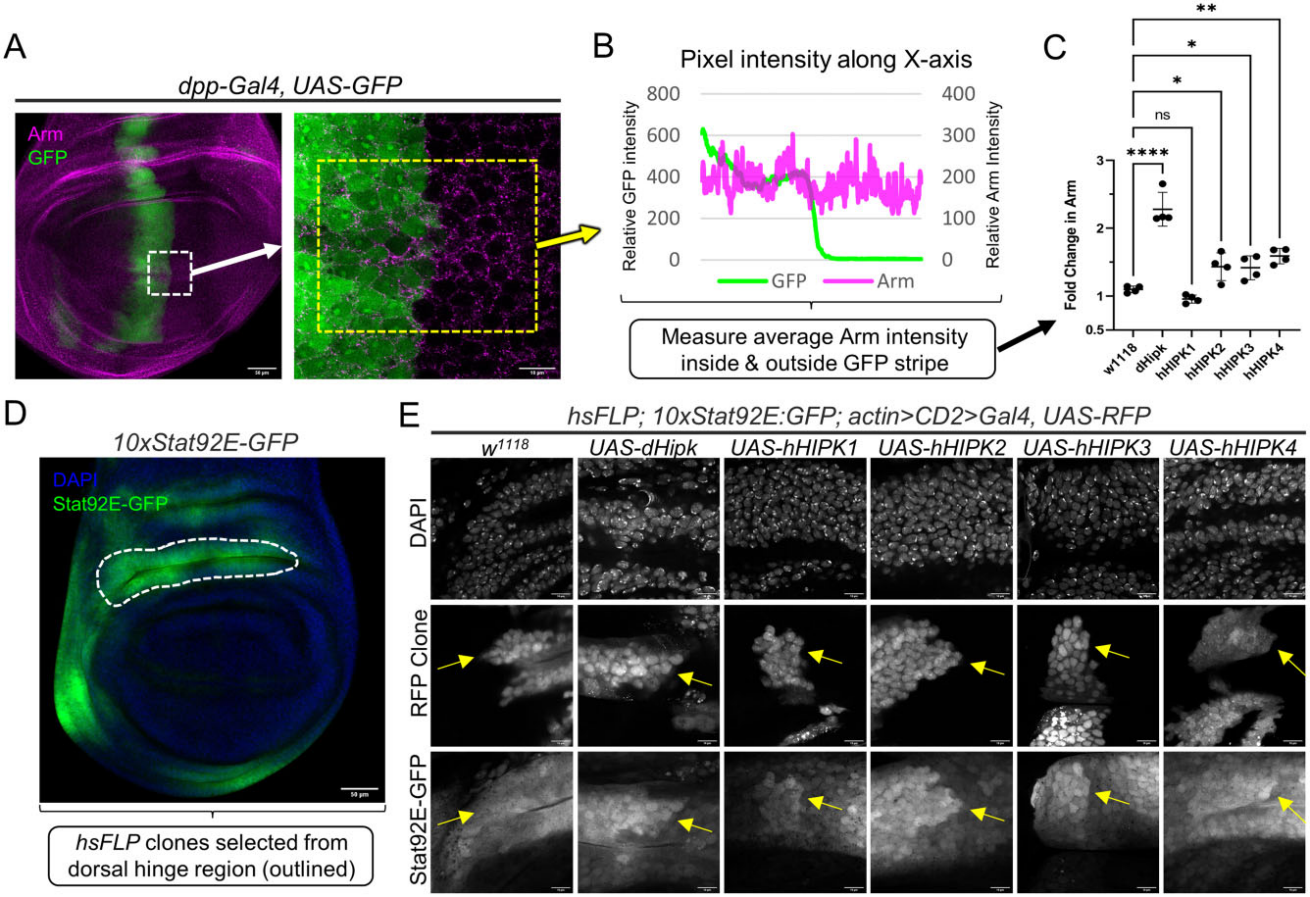
Human HIPKs phenocopy dHipk pathway alterations. (A) Representative image of a 3^rd^ instar wing imaginal disc control sample expressing GFP in the *dpp-Gal4* domain, and counterstained for Armadillo (Arm). The left image is a zoomed-out version of the image on the right, meant to provide context to our region of interest, highlighted in the dashed white box. The image on the right is zoomed in to focus on the region where the *dpp-Gal4* domain (marked with GFP) intersects the dorsal-ventral boundary of the wing disc that contains stabilized Arm. (B) Four samples were imaged at this magnification per cross, and the pixel intensity across the *x*-axis of the region within the dashed yellow box (as shown in panel A) was measured using ImageJ and plotted. For each image, a graph was generated to define the region of GFP and transgene expression along the *x*-axis. Once defined, the average pixel intensity of Arm was measured across these regions. (C) The average pixel intensity of Arm in the indicated genotypes was measured using the method shown in panel B. To calculate the fold change in Arm, the Arm signal for the GFP positive region was divided by that of the GFP negative region. Error bars indicate the mean with a 95% confidence interval. A one-way ANOVA was performed followed by Dunnett’s test to correct for multiple comparisons to *w1118* for each dataset. *P*-values for the statistical analyses performed correspond to the following symbols: ≥0.0332 (ns), <0.0332 (*), <0.0021(**), <0.0002(***), < 0.0001(****) (D) 3rd instar imaginal wing disc expressing the JAK/STAT pathway reporter *10xStat92E-GFP*, co-stained for DNA (DAPI) in blue to highlight tissue morphology. The dorsal hinge region of the wing disc, surrounded in the dashed white line, was used in our assessment of HIPKs on JAK/STAT activity. (E) Flp-out clones of cells expressing UAS-Hipks were generated in the dorsal hinge region defined in Figure 2D. Clones were marked in RFP, with DAPI acting as a counterstain. Yellow arrows indicate RFP clone edges and the corresponding tissue areas showing *10xStat92E-GFP* reporter expression. All images are from crosses performed at 29°C.

Next, we were interested in comparing the ability of human HIPKs to modulate specific signaling pathways known to be affected by dHipk. Our group has previously shown that dHipk and vertebrate HIPK2 are able to increase the stability of the key Wnt/Wingless effector protein Armadillo/β-Catenin by inhibiting its ubiquitin-mediated degradation (Lee *et al.* 2009b; Swarup and Verheyen 2011). Therefore, we assessed the ability of human HIPKs to stabilize endogenous Armadillo (Arm) in *Drosophila* by expressing HIPKs using *dpp-Gal4*, which drives transgene expression in a small stripe of cells along the anterior-posterior boundary of the developing wing imaginal disc (Figure 2A). Arm is expressed ubiquitously and is enhanced in two stripes flanking the dorsal-ventral boundary of the wing disc due to high levels of Wingless signaling (Peifer *et al.* 1994). We quantified pixel intensity to compare Arm levels in cells expressing transgenes and in flanking wild-type cells (Figure 2B). Consistent with our previous results with dHipk, we found that hHIPK2, hHIPK3, and hHIPK4 expression significantly increased the amount of Arm at the dorsal-ventral boundary of wing imaginal discs, while hHIPK1 was unable to do so (Figure 2C).

Our group has recently demonstrated that dHipk is required for JAK/STAT signaling during *Drosophila* development (Tettweiler *et al.* 2019). We therefore assessed the ability of the four human HIPKs to enhance JAK/STAT signaling in the hinge region of the wing imaginal disc where endogenous JAK/STAT signaling is most prominent (Figure 2D). We used the *10xSTAT92E-GFP* reporter containing ten STAT92E binding sites driving expression of an EGFP cDNA to provide a readout of JAK/STAT pathway activity (Bach *et al.* 2007). We generated random UAS-transgene expressing clones using the flp-out technique as described in the methods. RFP expression marks clones in which UAS-transgenes are expressed. We found that each of the four hHIPKs and dHipk variably caused an increase in endogenous JAK/STAT activity in clones found in the hinge region of the wing imaginal disc (Figure 2E, arrows).

### hHIPKs variably induce cell death and proliferation

Hipks have been shown to have variable and conflicting abilities to promote cell proliferation, tissue growth, and apoptosis through modulation of signaling pathways (Blaquiere and Verheyen 2017). Using a different *UAS-dhipk* insertion strain (*UAS-Hipk^3M^*) which has higher expression levels than the *attP40* strain used in this work promotes cell proliferation and tissue growth in the wing imaginal disc (Blaquiere *et al.* 2018; Wong *et al.* 2019, 2020). Therefore, we tested the ability of dHipk and hHIPKs to promote cell proliferation, tissue growth, and apoptosis in those same assays.

**Figure 3:**
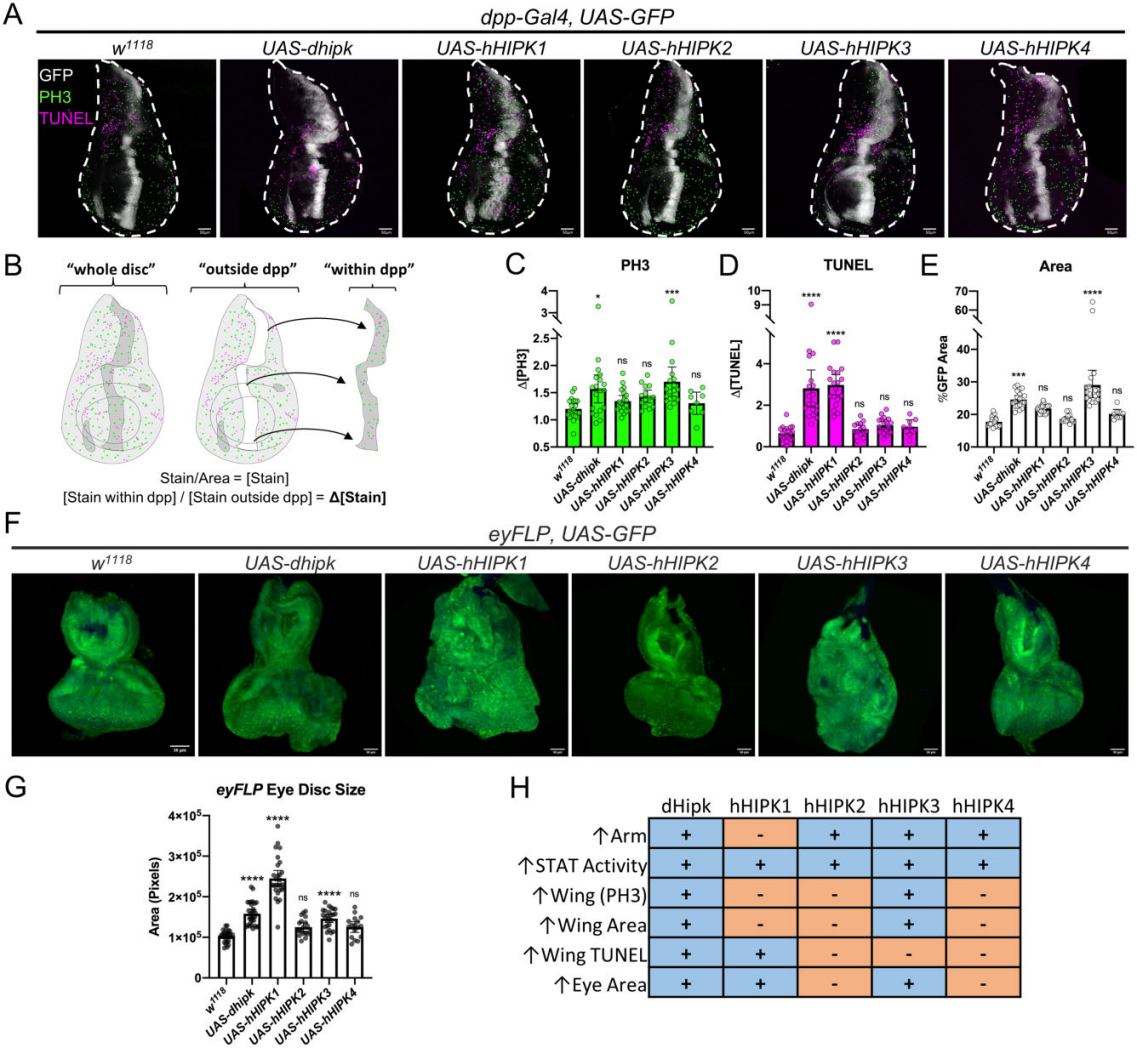
Human HIPKs variably induce cell proliferation, apoptosis, and tissue growth. (A) Representative images of 3rd instar imaginal wing discs of the corresponding genotypes stained for the mitotic marker PH3 (green) and the apoptosis marker TUNEL (magenta), with GFP (white) marking the *dpp-Gal4* domain where *UAS* constructs are expressed. Scale bars are 50 µm. (B) Diagram explaining how changes in PH3 and TUNEL stains were quantified. (C–E) Graphs show the change in PH3 staining, TUNEL staining, and area caused by expression of *UAS-Hipk* constructs. (F) Representative images of 3^rd^ instar imaginal eye-antennal discs expressing *UAS-Hipks and UAS-GFP* using the *eyFLP* genetic construct that produces strong *UAS* transgene expression within the entire eye-antennal disc. (G) Graph depicting the area of eye-antennal discs measured using FIJI. For both wing and eye disc experiments, the Gal4 driver crossed to *w^1118^* was used as the control. (H) Summary table for data presented in Figures 2 and 3. For all graphs, error bars indicate the mean with a 95% confidence interval. Statistical analysis included a one-way ANOVA followed by Dunnett’s test to correct for multiple comparisons to the control sample *w^1118^*. *P*-values for the statistical analyses performed correspond to the following symbols: ≥0.0332 (ns), <0.0332 (*), <0.0021(**), <0.0002(***), < 0.0001(****). Scale bars in representative images are 50 µm. Flies were raised at 29°C.

Using *dpp-Gal4* to drive expression of *UAS-HIPKs* in combination with *UAS-GFP* to mark the expression domain, we imaged wing discs to detect the proliferation marker phosphorylated histone 3 (PH3), and performed terminal deoxynucleotidyl transferase dUTP nick end labeling (TUNEL) to detect apoptosis (Figure 3, A–E) (Gavrieli *et al.* 1992). Comparing the concentration of PH3 and TUNEL in GFP positive and GFP negative tissues let us determine how each HIPK affected cell proliferation and apoptosis (Figure 3, B–D). Similarly, measuring the area of the GFP domain compared to the overall wing disc area allowed us to measure HIPK-mediated changes to tissue growth (Figure 3, B and E). Expression of dHipk or hHIPK3 caused a significant increase in PH3 in the wing disc, while no change was detected when hHIPK1, hHIPK2, or hHIPK4 were expressed (Figure 3C). Similarly, only dHipk and hHIPK3 caused a significant increase in the tissue size (Figure 3E). Finally, we found that dHipk and hHIPK1 significantly induce apoptosis in the wing imaginal disc, as we had seen with dHipk previously (Figure 3D) (Blaquiere *et al.* 2018). Together, these data show that HIPKs variably induce proliferation, tissue growth, and apoptosis. To see if the effects of HIPKs changed when expressed in different tissues, we switched to using the *eyFLP* technique which causes high levels of Gal4 expression throughout the eye-antennal disc. In addition to dHipk and hHIPK3, hHIPK1 was also able to drastically increase tissue size, with a marked distortion of tissue morphology occurring when either hHIPK1 or hHIPK3 was expressed (Figure 3, F and G) (Pagliarini and Xu 2003). Thus, these experiments revealed that hHIPKs share many functions with dHipk, but not one single hHIPK was able to perform all dHipk functions in these assays (Figure 3H).

### hHIPK1 and hHIPK2 expression causes adult wing defects due to Ubx induction

**Figure 4:**
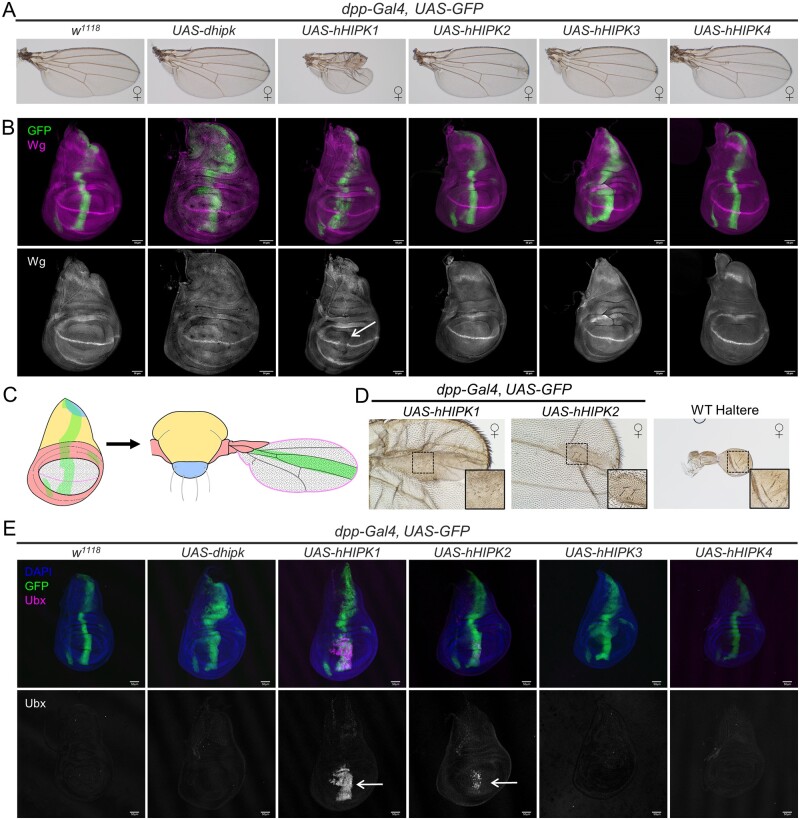
hHIPKs have distinct effects on wing patterning. (A) Representative adult wings dissected from the corresponding genotypes. (B) Representative images of late 3^rd^ instar imaginal wing discs dissected from larvae of corresponding genotypes and stained for Wg. Wing discs expressing *UAS-hHIPK1* show a loss of Wg staining at the dorsal-ventral boundary (arrow). (C) Graphical representation of the *dpp-Gal4* domain in larval wing disc and adult wing tissues. Green indicates the *dpp-Gal4* domain, while other colors and patterns indicate corresponding regions between the larval and adult wing. (D) Zoomed in image of *dpp-Gal4, UAS-hHIPK1 or UAS-hHIPK2* wing phenotype, compared to a wild-type haltere (images are to scale). Inset boxes for each image focus on similar phenotypes between the three images. (E) Representative images of late 3rd instar imaginal wing discs dissected from larvae of the corresponding genotypes and stained for the Hox protein Ubx. Wing discs expressing *UAS-hHIPK1* or *UAS-hHIPK2* show Ubx induction in the wing pouch (arrows). Results were consistent across 10 wing imaginal discs assessed for each genotype. (B,E) GFP marks the *dpp-Gal4* domain where *UAS* constructs are expressed. For all images, the sex of the representative tissues was picked from mixed-sex samples unless otherwise noted by the female (♀) symbol. All crosses were performed at 29°C.

**Figure 5:**
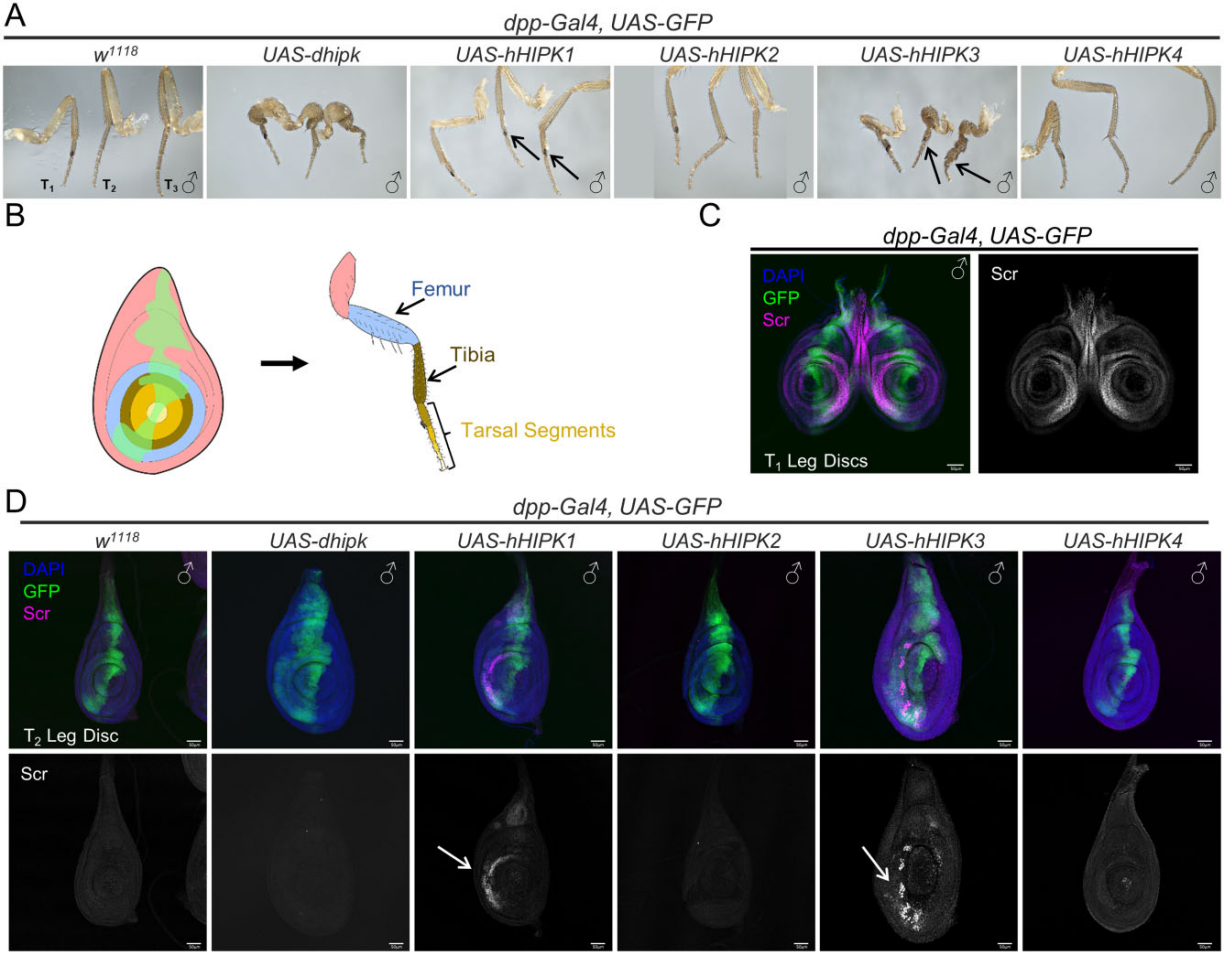
Leg development is differentially affected by expression of Hipks. (A) Representative adult male prothoracic (T_1_), mesothoracic (T_2_), and metathoracic (T_3_) legs dissected from the corresponding genotypes. Arrows indicate ectopic sex combs. (B) Graphical representation of the *dpp-Gal4* domain in larval leg imaginal disc and adult leg tissues. Green indicates the *dpp-Gal4* domain, while other colors indicate corresponding regions between the larval and adult leg. (C) Image of control late 3rd instar prothoracic (T_1_) imaginal leg discs stained for the Hox protein Scr. (D) Representative images of late 3rd instar mesothoracic (T_2)_ imaginal leg discs dissected from larvae of the corresponding genotypes and stained for the Hox protein Scr. Results were consistent across 10 T_2_ imaginal leg discs assessed for each genotype. GFP marks the *dpp-Gal4* domain where *UAS* constructs are expressed. All adult and larval flies assessed in this figure were male. Crosses were performed at 29°C. (C,D) Scale bars: 50µm.

The experiments performed above highlight the diversity of shared and unique functions of HIPKs. To further address which activities individual HIPKs can perform, we monitored adult phenotypes resulting from ectopic expression of the hHIPKs in a wildtype background. We used the *dpp-Gal4* driver, which has well-defined and discrete expression patterns in the developing wing and leg imaginal discs (Figures 4C and 5B) (Staehling-Hampton *et al.* 1994). As with the pathways assessed previously in Figures 2 and 3, these experiments were carried out at 29°C to promote obvious phenotypic changes due to high Gal4 transcriptional activity.

Expression of *UAS-hHIPK1* or *UAS-hHIPK2* caused patterning abnormalities of the adult wing when expressed using *dpp-Gal4* (Figure 4A). *UAS-hHIPK1* caused severe wing notching and vein abnormalities, while *UAS-hHIPK2* caused abnormalities to the distal central region of the wing blade, corresponding to the domain where dpp-Gal4 is expressed (Figure 4C). Wing notching is characteristic of either disrupted Notch or Wingless signaling. Notch is required for the expression of Wingless (Wg) at the dorsal-ventral boundary, the region that specifies the edge of the adult wing blade (Rulifson and Blair 1995). The *dpp-Gal4* expression pattern in the wing disc crosses through the region that produces the adult wing margin (Figure 4C). We therefore stained 3^rd^ instar wing imaginal discs to detect Wg while expressing each of the hHIPKs or dHipk. Wing imaginal discs expressing *UAS-hHIPK1* had reduced Wg staining where *dpp-Gal4* intersects the dorsal-ventral boundary (Figure 4B, arrow). Flies expressing *UAS-hHIPK2* show milder wing defects and appeared to have intact Wg staining, as did wing discs expressing the other Hipks.

Upon closer inspection, the region of the adult wing expressing either *UAS-hHIPK1* or *UAS-hHIPK2* contained altered wing pigmentation, as well as small hairs and sensory bristles not normally found on the wing, instead resembling those found on the rudimentary hind wing-like structures called halteres (Figure 4D). The altered development of wing tissue causing it to fully or partially develop into haltere tissue is a homeotic transformation commonly associated with the misexpression of the homeobox (Hox) gene *Ubx* (Weatherbee *et al.* 1998; Pearson *et al.* 2005). Furthermore, Ubx misexpression is known to inhibit Notch’s ability to regulate Wg expression at the dorsal-ventral boundary of the wing imaginal disc (Weatherbee *et al.* 1998). Therefore, we asked whether the phenotypes we observed could be due to ectopic Ubx in wing discs. We found that expression of either *UAS-hHIPK1* or *UAS-hHIPK2* caused ectopic induction of Ubx in the wing pouch, but not in other regions of the wing disc where *dpp-Gal4* is expressed (Figure 4E). The degree of Ubx induction was greater in wing discs expressing hHIPK1 compared to those expressing hHIPK2, which matches the severity of the adult wing phenotypes. Together, these data suggest that hHIPK1 and hHIPK2 each induce ectopic *Ubx* expression in the wing pouch, resulting in a wing-to-haltere homeotic transformation.

### hHIPK1 and hHIPK3 expression causes deformed legs, and Scr-induced ectopic sex combs

We have previously demonstrated that expression of high levels of *UAS-dhipk* in the leg using *dpp-Gal4* causes malformed adult legs due to aberrant proliferation (Figure 5A) (Wong *et al.* 2020). We therefore tested the effects of expressing *UAS-hHIPKs* in a discrete domain in the leg disc using *dpp-Gal4* (Figure 5B). Only *UAS-hHIPK3* caused severely malformed legs like those seen with dHipk, while *UAS-hHIPK1* caused less severe malformations (Figure 5A, Supplementary Table S1). In addition, we found that both *UAS-hHIPK1 and UAS-hHIPK3* caused ectopic sex comb formation on the middle and rear legs of males, where they are not normally found (Figure 5A, arrows, Supplementary Table S1). *dpp-Gal4* is expressed in the region that gives rise to the sex combs in the leg imaginal discs (Figure 5B). The specification of sex combs requires the expression of the Hox protein Sex combs reduced (Scr) as seen in a control pair of the first set of leg discs called T_1_ (Figure 5C). Scr is absent in wild-type middle legs (T_2_; Figure 5D). We stained the T_2_ leg discs with anti-Scr antibodies and found that those expressing *UAS-hHIPK1* or *UAS-hHIPK3* consistently showed ectopic Scr expression (arrows in Figure 5D). We also observed that hHIPK1 alone was able to cause loss of the antennal bristle called the arista (Supplementary Figure S3). Such an aristaless phenotype has been described as a minor antenna-to-leg transformation (Sadasivam and Huang 2016). While the Hox protein Antennapedia (Antp) is frequently found to be ectopically expressed in eye-antennal imaginal discs that undergo antenna-to-leg transformations, we did not observe this (data not shown) (Struhl 1981). However, partial antenna-to-leg transformations such as what we observed can occur without detectable levels of Antp, suggesting that Antp may be below the level of detection in our assay (Sadasivam and Huang 2016). Thus, hHIPKs are capable of driving ectopic expression of at least two Hox genes, *Ubx and Scr*, in discrete domains in specific discs.

Mutations in components of the Polycomb group complexes (PcGs), which impart epigenetic gene regulation during development, are known to result in misexpression of Hox genes in larval imaginal discs (Kassis *et al.* 2017). The Hox genes that are mis-expressed in PcG mutants are often specific to different tissues, with Ubx mis-expressed in the wing imaginal disc, and Scr in the leg imaginal discs, similar to what we have observed with hHIPK expression using the *dpp-Gal4* driver. There is evidence for individual mutants of PcG component genes to produce different severity of *Hox* misexpression that depends on which component is mutated, with differences in the intensity and tissue region of ectopic *Hox* induction. One example provided by Beuchle *et al.*, 2001, demonstrated the variable induction of *Ubx and Abdominal-B* (*Abd-B*) concomitant with individual PcG mutants in the wing imaginal disc (Beuchle *et al.* 2001). We therefore stained larval tissues expressing *UAS-hHIPKs* to detect AbdB and found that *UAS-hHIPK1* alone was able to induce ectopic AbdB expression in wing, leg, and eye-antennal imaginal discs (Supplementary Figure S4, A–C). Of note, the tissue regions where AbdB was induced in wing or leg imaginal discs were different compared to the domains where Ubx or Scr, respectively, were induced by hHIPK1.

## Discussion

Vertebrate Hipks are necessary for normal development, however much remains to be learned about their individual functions. Our incomplete understanding of the four vertebrate Hipks is exacerbated by functional redundancy, which has made it difficult to adequately study their comparative roles with individual knockouts. While cell culture studies have contributed to our understanding of Hipk functions, no work has been done to compare the ability of the four vertebrate Hipks to modulate developmental pathways in vivo. Unlike vertebrates, *Drosophila* has only a single dHipk that can perform many of the same functions described for vertebrate Hipks. The fly *dhipk* can also be easily knocked out, with ectopic expression of transgenic vertebrate Hipks in its place. We therefore used the fly to compare the functions of the four human HIPKs. Our results provide three key comparisons and insights. First, our rescue experiments demonstrated the extent to which each of the human HIPKs can functionally replace *Drosophila* Hipk for survival and morphological development. Second, we demonstrated the ability of each human HIPK to modulate Arm levels, JAK/STAT activity, proliferation, growth, and death, each of which have previously been described for Hipks, but never all together in comparable tissues. Third, we characterized novel phenotypes induced by human HIPKs to gain insight to their unique functions. Together, these experiments provide a direct comparison of all four vertebrate HIPKs to determine if they are capable of performing the same roles in a developmental model.

Our rescue experiments were designed to test the ability of human Hipks to rescue or suppress the pupal lethality found in *dhipk* mutant flies. Expression of hHIPKs using Gal4 inserted in the *hipk* locus revealed that hHIPK1 and hHIPK2 each rescue *dhipk* mutant lethality, while hHIPK3 and hHIPK4 cannot. The ability of these human HIPKs to rescue *dhipk* mutants shows that they possess conserved functions. This is consistent with work from Isono *et al.*(2006), where Hipk1 and Hipk2 were shown to have overlapping roles during mouse development by analysis of double *Hipk1/Hipk2* knockouts (Isono *et al.* 2006). However, their work did not assess the possibility of functional redundancy between Hipk3 or Hipk4. The inability of hHIPK3 or hHIPK4 to rescue *dhipk* mutant lethality in our work suggests that their roles are more divergent from those of hHIPK1 and hHIPK2, or that they are regulated differently. This is not surprising for hHIPK4, since it lacks nearly all similarities to hHIPK1, hHIPK2, and dHipk outside of the kinase domain (Figure 1A), however hHIPK3 is highly similar to these Hipks, so its inability to rescue *dhipk* mutant lethality may warrant further investigation into the significance of the amino acid sequence differences between these proteins.

The rescue of *dhipk* mutant lethality by hHIPK1 and hHIPK2, but not hHIPK3 or hHIPK4, provides new information in our understanding of comparative Hipk functions, however it does not tell the whole story. hHIPK2 not only rescued lethality, but also each of the head defects caused by *dhipk* knockout, which shows that it can perform multiple similar functions to dHipk. In comparison to hHIPK2, the ability of hHIPK1 to rescue lethality, but not any of the head defects suggests that the functions controlling lethality in the fly are distinct from those that regulate eye, ocellar, and bristle development. The idea of separate functions is further highlighted by the inability of hHIPK3 and hHIPK4 to rescue lethality while still rescuing ocellar and bristle loss, respectively, demonstrating that the four hHIPKs have varying abilities to perform dHipk functions. It is not clear to us how hHIPK2 was better at rescuing the *dhipk* mutant than dHipk itself was. We speculate that the deleterious effects of dHipk overexpression need to be balanced with the restoration of essential functions, and that this delicate balance is hard to achieve. However, the fact that the HIPKs are all able to affect JAK/STAT signaling to varying degrees indicates that each of the hHIPKs are functioning adequately in the fly.

In our work, we examined the abilities of human Hipks to carry out functions that have been established for dHipk. In these studies, we expressed hHIPKS in a wildtype genetic background, and assayed a number of readouts of dHipk activity. Among these, we examined the modulation of Wnt/Wingless and JAK/STAT signaling, as well as cell proliferation, tissue growth, and apoptosis which are controlled by multiple signaling pathways, many of which are modulated by Hipks (Link *et al.* 2007; Lee *et al.* 2009b; Swarup and Verheyen 2011; Poon *et al.* 2012; Blaquiere *et al.* 2018; Tettweiler *et al.* 2019). Our findings revealed that human Hipks have distinct roles that are consistent with the vertebrate literature, and that none behaves exactly like dHipk, which is not unexpected. The roles of vertebrate Hipks in cell proliferation and tissue growth are conflicting and very context dependent (Blaquiere and Verheyen 2017). We found that dHipk and hHIPK3 increase proliferation and tissue growth in wing imaginal discs, while dHipk, hHIPK1, and hHIPK3 increase tissue growth in eye-antennal discs, suggesting distinct functions in different tissues. We previously found that high level expression of dHipk could induce both proliferation and apoptosis (Blaquiere *et al.* 2018) and in this work, we found dHipk and hHIPK1 expression led to increased apoptosis. Of note, hHIPK2, HIPK3, and HIPK4 did not increase apoptosis. These findings were notable given the well-established role for HIPK2 in promoting apoptosis. However, HIPK2 has only been described to promote p53-mediated apoptosis in conditions of cellular or genotoxic stress (D’Orazi *et al.* 2002; Hofmann *et al.* 2002). To date, the roles of HIPK3 and HIPK4 in stress-induced death are not well understood. Our experiments were not designed to promote such stresses, which may explain the absence of apoptosis when hHIPKs 2, 3, and 4 were expressed. In contrast, the ability of dHipk and hHIPK1 to induce apoptosis in the absence of cellular or genotoxic stress suggests that they use a distinct mechanism.

The ability of each of the human HIPKs to increase JAK/STAT signaling, as revealed by a STAT-responsive reporter, shows that this Hipk function is conserved across homologs, and it also indicates that this function is at least partially performed in the cytoplasm, since hHIPK4 is only found in the cytoplasm, while the other HIPKs can shuttle between the nucleus and cytoplasm (Moller *et al.* 2003; Arai *et al.* 2007; Van der Laden *et al.* 2015). HIPK2 was previously shown to be able to phosphorylate STAT3, which suggests other hHIPKs may also play such roles in vertebrates (Matsuo *et al.* 2001).

The ability of hHIPK2, hHIPK3, and hHIPK4 to increase Arm levels in the wing disc is likely due to previously described cytoplasmic activity of HIPKs, where dHipk and Hipk2 were shown to inhibit the ubiquitin ligase that targets Arm/β-Catenin for degradation (Swarup and Verheyen 2011). HIPK1 was an outlier since it did not lead to Arm stabilization. This is most likely due to the fact that Wg protein expression is suppressed by hHIPK1, and Wg is required for Arm stabilization in signal receiving cells. One explanation for reduced Wg was the finding that hHIPK1 could induce high levels of ectopic Ubx protein. Ubx inhibits Notch signaling, thereby preventing expression of the Notch target *wingless* (Weatherbee *et al.* 1998). We also found that hHIPK2 causes a mild upregulation of Ubx, which could lead to reductions in Wg that were not detectable at this level of resolution, but which were apparent from the lower level of Arm stabilization, compared to the effects of hHIPK3 or hHIPK4.

In the overexpression experiments, we made a novel set of observations that expression of hHIPK1-3 could lead to homeotic transformations, or homeosis. In each case, we found the ectopic expression of a particular Hox protein in strict tissue domains, which suggests a highly regulated process. For example, Ubx was induced only in the wing pouch region of the wing disc (Figure 4E), even though hHIPK1 was expressed in a broader domain in that disc, and no Ubx was observed in leg or eye imaginal discs, despite hHIPK1 expression in those tissues.

Hipk proteins were named for their initial discovery as binding partners of proteins containing homeodomains which are generally involved in transcriptional regulation. While several studies have found direct protein–protein interactions between Hipks and homeodomain-containing proteins such as Eyeless/Pax6 and NK3 (Kim *et al.* 1998, 2006; Choi *et al.* 1999; Steinmetz *et al.* 2018), it is important to note that the homeotic transformation phenotypes we observed following hHIPK expression are not indicative of direct interaction with Hox proteins. Instead, the homeotic transformations observed in these experiments are well-characterized phenotypes associated with the upregulation of *Hox* gene transcription (Kassis *et al.* 2017). Thus, Hipks appear to play dual roles with Hox proteins, in regulating their transcription (albeit indirectly, see below) and through protein–protein interactions regulating their activity.

Homeotic transformations are well-studied phenomena found to occur due to mutations in *Hox* genes or dysregulation of chromatin regulating complexes. Trithorax group (TrxG) and Polycomb group (PcG) complexes are two opposing types of chromatin-modifiers that epigenetically regulate *Hox* gene expression (Lau and So 2015). TrxG proteins promote target gene expression, while PcG proteins repress transcription through differential histone methylation. The ability of HIPKs 1, 2, and 3 to induce ectopic *Hox* gene expression, causing homeotic transformations, is very similar to what happens when PcG function is disrupted, or TrxG activity is enhanced (Sadasivam and Huang 2016; Kassis *et al.* 2017). It is therefore tempting to speculate that hHIPKs function to either promote the activity of the TrxG complex or repress the activity of PcG complexes. There is support for this model, since HIPK2 can associate with the Polycomb protein Pc2/CBX4, which is part of the Polycomb repressive complex 1 (PRC1) (Roscic *et al.* 2006). Another recent study used HIPK2 tethered to chromatin to directly address its ability to modulate chromosome compaction, where chromatin bound HIPK2 led to decreased Histone 3 Lysine 27 trimethylation (H3K27me3), an epigenetic mark normally associated with transcriptional repression and formation of heterochromatin (Haas *et al.* 2020). Thus, our findings of phenotypes associated with dysfunction of TrxG/PcG and previous work suggest that Hipks may play roles in regulating chromatin condensation. While we are not sure how the individual HIPKs induce different homeotic transformations and ectopic *Hox* expression, this will be an interesting area of future study, since PcG regulators are extremely important in development, and valuable to understand mechanistically in cancer (Sauvageau and Sauvageau 2010).

This study collectively shows that Hipks share many conserved functions across species and validates the use of *Drosophila* as a tool to understand this complex and multi-facetted kinase family. Furthermore, our findings reveal intriguing potential roles for hHIPKs in chromatin dynamics.

## Data availability

Fly strains and reagents are available upon request.

Supplementary material is available at *G3* online.

## Supplementary Material

jkab350_Supplementary_Figure-Tables

jkab350_Supplementary_Figure-Tables-Captions
