# Supplementary material for: Expression of human HIPKs in Drosophila demonstrates their shared and unique functions in a developmental model
Source: G3 (Bethesda). 2021 Oct 4;11(12):jkab350. doi: 10.1093/g3journal/jkab350 (PMC8673556; doi:10.1093/g3journal/jkab350)
Supplement: jkab350_Supplementary_Figure-Tables [file jkab350_supplementary_figure-tables.pdf]

Fig S1

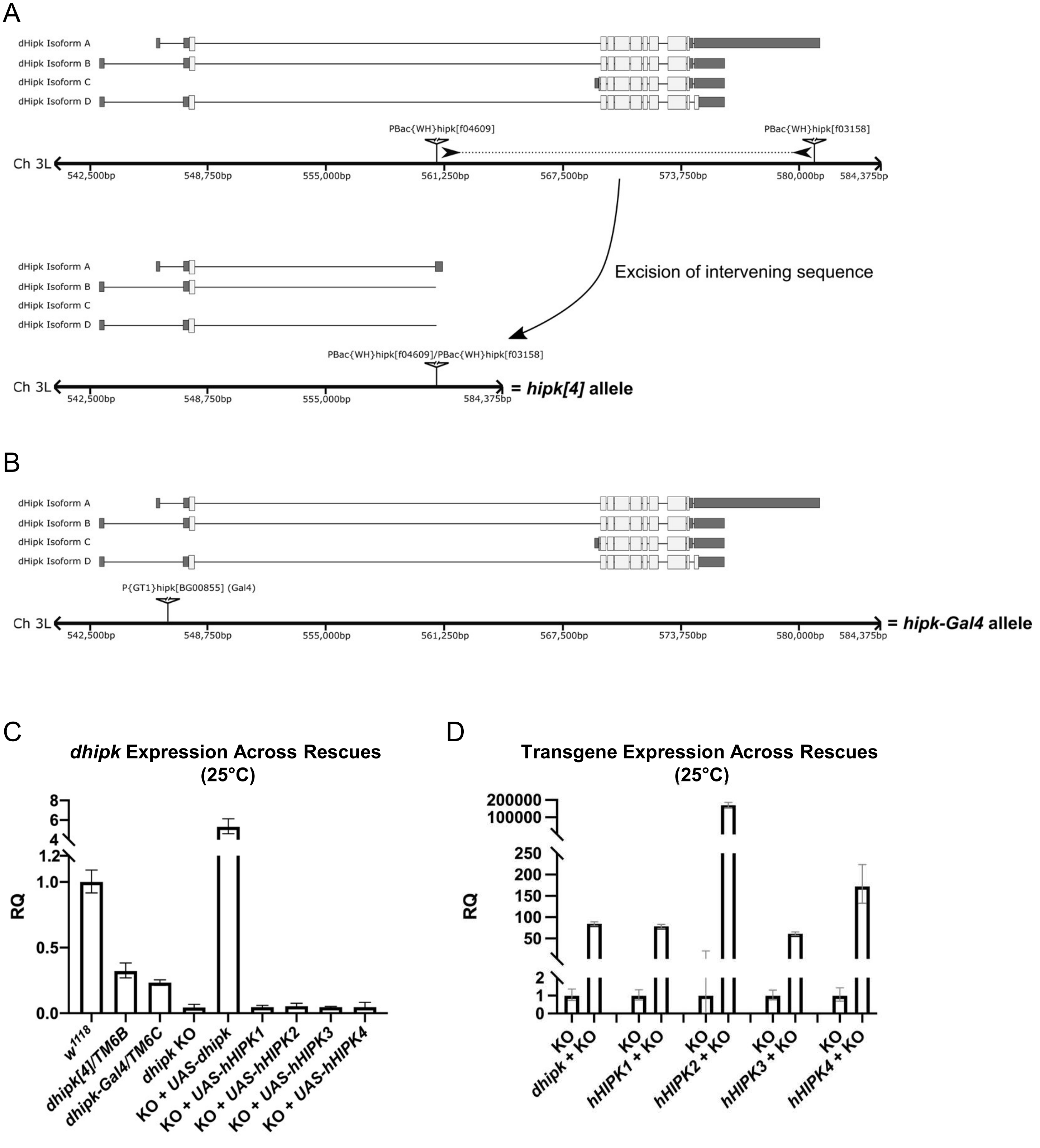
A
dHipk Isoform A
dHipk Isoform B
dHipk Isoform C
dHipk Isoform D
PBac{WH}hipk[f04609]
PBac{WH}hipk[f03158]
Ch 3L
542,500bp
548,750bp
555,000bp
561,250bp
567,500bp
573,750bp
580,000bp
584,375bp
Excision of intervening sequence
PBac{WH}hipk[f04609]/PBac{WH}hipk[f03158]
= hipk[4] allele
B
P{GT1}hipk[BG00855] (Gal4)
= hipk-Gal4 allele
C
dhipk Expression Across Rescues (25°C)
RQ
w1118
dhipk[4]/TM6B
dhipk-Gal4/TM6C
dhipk KO
KO + UAS-dhipk
KO + UAS-hHIPK1
KO + UAS-hHIPK2
KO + UAS-hHIPK3
KO + UAS-hHIPK4
D
Transgene Expression Across Rescues (25°C)
RQ
KO
dhipk + KO
hHIPK1 + KO
hHIPK2 + KO
hHIPK3 + KO
hHIPK4 + KO

Fig S2

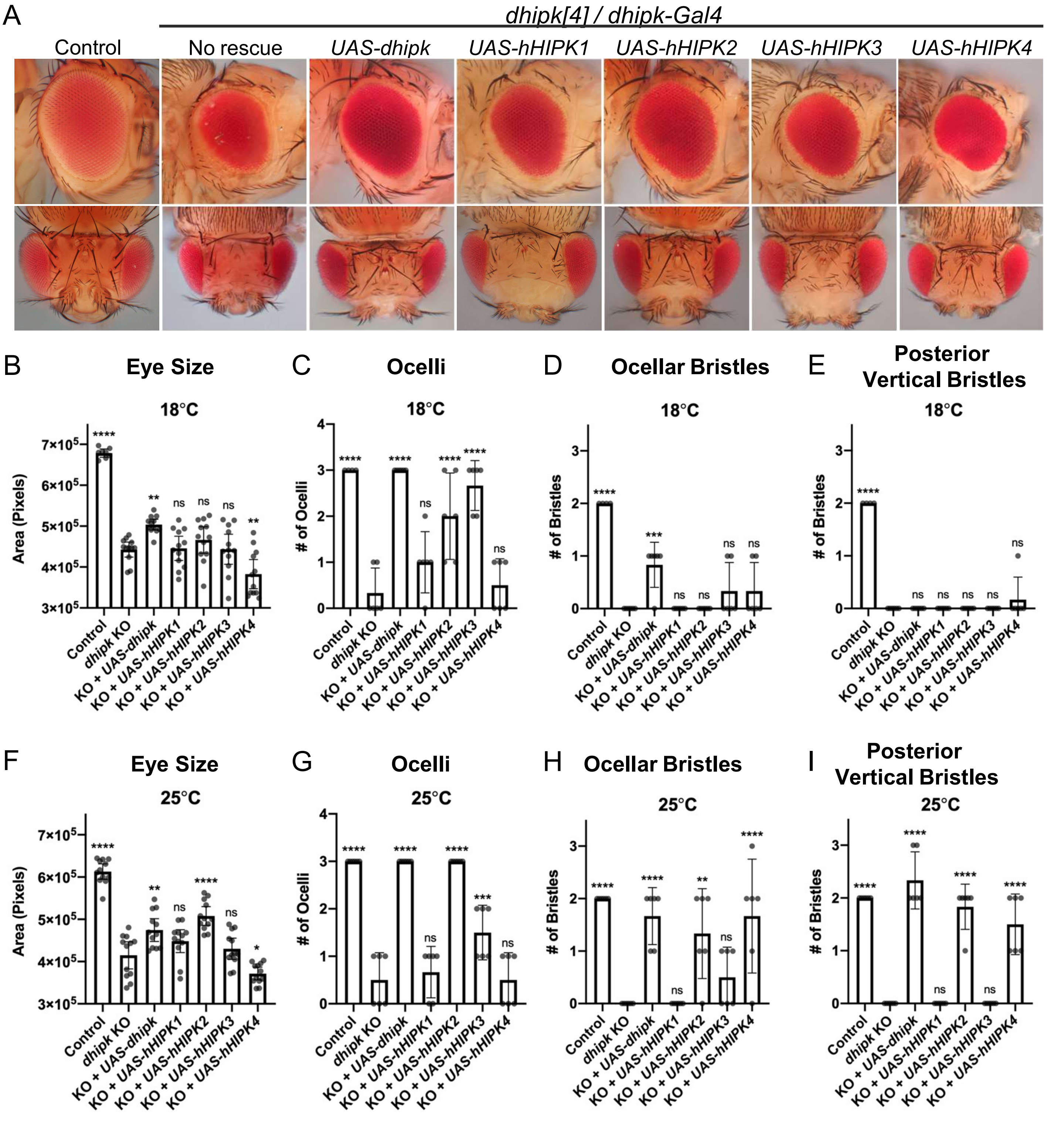
A
dhipk[4] / dhipk-Gal4
Control
No rescue
UAS-dhipk
UAS-hHIPK1
UAS-hHIPK2
UAS-hHIPK3
UAS-hHIPK4
B
Eye Size
18°C
Area (Pixels)
7×10^5
6×10^5
5×10^5
4×10^5
3×10^5
****
**
ns
ns
ns
**
C
Ocelli
18°C
# of Ocelli
****
****
ns
****
****
ns
D
Ocellar Bristles
18°C
# of Bristles
****
***
ns
ns
ns
ns
E
Posterior Vertical Bristles
18°C
# of Bristles
****
ns
ns
ns
ns
ns
F
Eye Size
25°C
Area (Pixels)
****
**
ns
****
ns
*
G
Ocelli
25°C
# of Ocelli
****
****
****
ns
***
ns
H
Ocellar Bristles
25°C
# of Bristles
****
****
ns
**
ns
****
I
Posterior Vertical Bristles
25°C
# of Bristles
****
****
ns
****
ns
****
Control
dhipk KO
KO + UAS-dhipk
KO + UAS-hHIPK1
KO + UAS-hHIPK2
KO + UAS-hHIPK3
KO + UAS-hHIPK4

Fig S3

A

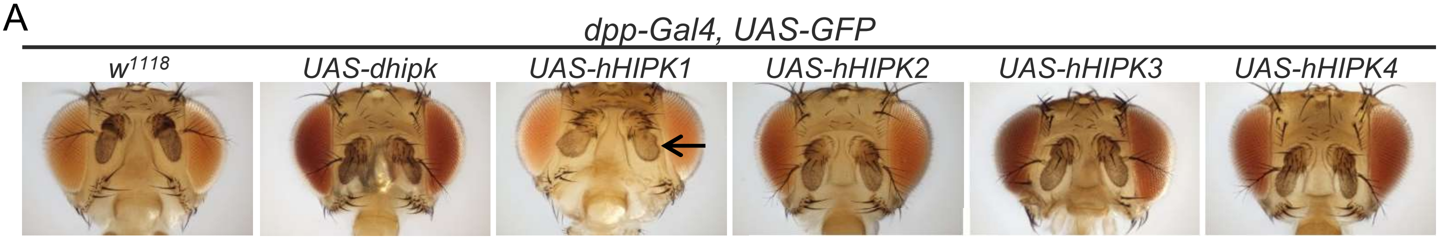

dpp-Gal4, UAS-GFP
w1118
UAS-dhipk
UAS-hHIPK1
UAS-hHIPK2
UAS-hHIPK3
UAS-hHIPK4

B

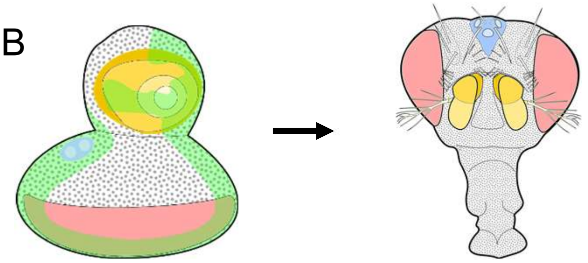

Fig S4

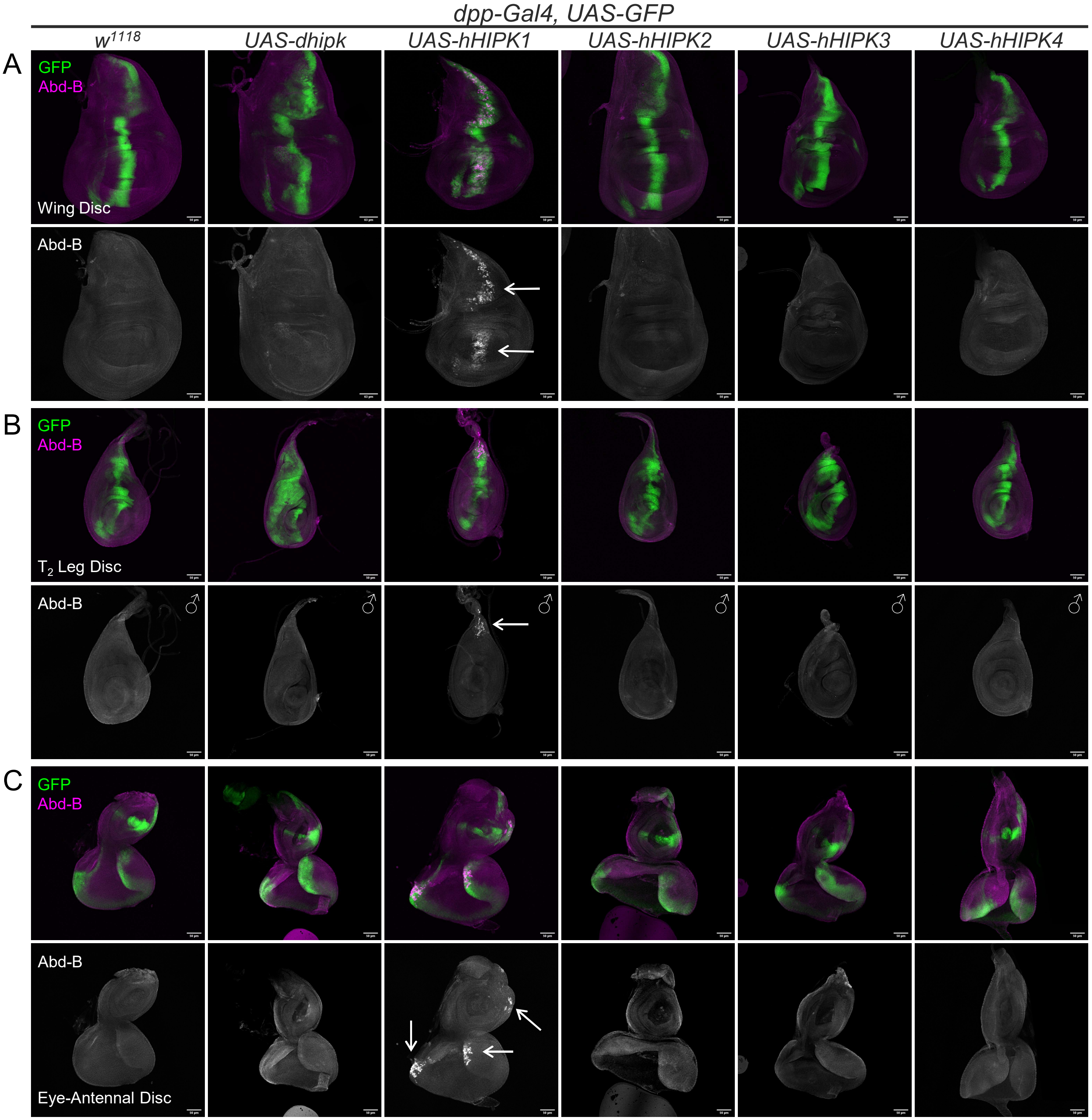
dpp-Gal4, UAS-GFP
w1118
UAS-dhipk
UAS-hHIPK1
UAS-hHIPK2
UAS-hHIPK3
UAS-hHIPK4
A
GFP
Abd-B
Wing Disc
Abd-B
B
GFP
Abd-B
T2 Leg Disc
Abd-B
♂
C
GFP
Abd-B
Abd-B
Eye-Antennal Disc

Fig S5

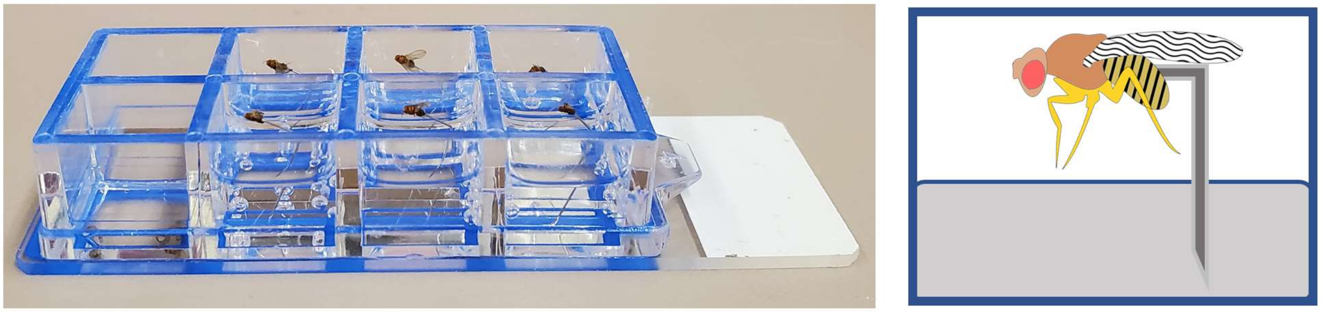

Table S1

**Genotype**	**# Legs Assessed**	**Deformed (by segment)**				**Sex Combs**
			Fe	Ti	Ta	
dpp-Gal4, UAS-GFP<br>***w***[***1118***]	$\mathbf{T_1}$: 7 / $\mathbf{T_2}$: 7 / $\mathbf{T_3}$: 7	T_1	0%	0%	0%	$\mathbf{T_1}$: 100% / $\mathbf{T_2}$: 0% / $\mathbf{T_3}$: 0%
		T_2	0%	0%	0%	
		T_3	0%	0%	0%	
			Fe	Ti	Ta	
dpp-Gal4, UAS-GFP<br>***UAS-dhipk***	$\mathbf{T_1}$: 14 / $\mathbf{T_2}$: 14 / $\mathbf{T_3}$: 13	T_1	100%	100%	0%	$\mathbf{T_1}$: 100% / $\mathbf{T_2}$: 0% / $\mathbf{T_3}$: 0%
		T_2	100%	100%	0%	
		T_3	100%	100%	0%	
			Fe	Ti	Ta	
dpp-Gal4, UAS-GFP<br>***UAS-hHIPK1***	$\mathbf{T_1}$: 21 / $\mathbf{T_2}$: 17 / $\mathbf{T_3}$: 19	T_1	57.1%	33.3%	61.9%	$\mathbf{T_1}$: 100% / $\mathbf{T_2}$: 88.2% / $\mathbf{T_3}$: 78.9%
		T_2	5.9%	5.9%	52.9%	
		T_3	57.9%	84.2%	57.9%	
			Fe	Ti	Ta	
dpp-Gal4, UAS-GFP<br>***UAS-hHIPK2***	$\mathbf{T_1}$: 19 / $\mathbf{T_2}$: 19 / $\mathbf{T_3}$: 18	T_1	0%	0%	0%	$\mathbf{T_1}$: 100% / $\mathbf{T_2}$: 0% / $\mathbf{T_3}$: 0%
		T_2	0%	0%	0%	
		T_3	0%	0%	0%	
			Fe	Ti	Ta	
dpp-Gal4, UAS-GFP<br>***UAS-hHIPK3***	$\mathbf{T_1}$: 15 / $\mathbf{T_2}$: 15 / $\mathbf{T_3}$: 18	T_1	80.0%	80%	20%	$\mathbf{T_1}$: 100% / $\mathbf{T_2}$: 80.0% / $\mathbf{T_3}$: 44.4%
		T_2	86.7%	73.3%	20%	
		T_3	94.4%	88.9%	38.9%	
			Fe	Ti	Ta	
dpp-Gal4, UAS-GFP<br>***UAS-hHIPK4***	$\mathbf{T_1}$: 19 / $\mathbf{T_2}$: 19 / $\mathbf{T_3}$: 18	T_1	0%	0%	0%	$\mathbf{T_1}$: 100% / $\mathbf{T_2}$: 0% / $\mathbf{T_3}$: 0%
		T_2	0%	0%	0%	
		T_3	0%	0%	0%	
