# Supplementary material for: Expression of human HIPKs in Drosophila demonstrates their shared and unique functions in a developmental model
Source: G3 (Bethesda). 2021 Oct 4;11(12):jkab350. doi: 10.1093/g3journal/jkab350 (PMC8673556; doi:10.1093/g3journal/jkab350)
Supplement: jkab350_Supplementary_Figure-Tables-Captions [file jkab350_supplementary_figure-tables-captions.docx]

**Supplemental Figure 1. *dhipk* mutant alleles and qPCR confirmation of knockout efficiency.** (A) The *dhipk[4]* allele was generated by P-element excision, as described previously [2]. (B) The *dhipk-Gal4* allele was generated in the Baylor genetrap screen by insertion of a P-element containing a Gal4 exon into the beginning of the *dhipk* gene [30]. (C) The expression of *dhipk* was compared between wild-type, heterozygous *dhipk* mutant, transheterozygous *dhipk* knockout, and *dhipk* knockouts expressing *UAS-Hipks*. (D) Expression of specific *hHIPKs* or *dhipk* was confirmed in the respective *dhipk* mutant rescue experiments using qPCR. For each *UAS-hHIPK* or *UAS-dhipk* rescue assessed, *dhipk* transheterozygous knockouts were used as the control. It is challenging to compare expression levels of human genes in *Drosophila* as there is no adequate control, in contrast to the *dhipk* transgene, which can be compared to the endogenous *dhipk* expression. Therefore, this figure is only intended to provide validation of correct transgene expression, not relative expression levels across transgenes. (C,D) Two male and two female 3^rd^ instar larvae from each cross raised at 25°C were used in these experiments. Bars represent the mean, while error bars represent the upper and lower limits as defined by Quantstudio Design and Analysis Software.

**Supplemental Figure 2. hHIPKs variably rescue *dhipk* mutant head phenotypes.**

(A) Representative heads and eyes from *dhipk* mutant flies expressing individual *UAS-hHIPKs* or *UAS-dHipk* using the *dhipk-Gal4* driver. Six flies were imaged for each cross. (B) The area of the eyes, (C) the number of ocelli, (D) the number of ocellar bristles, and (E) the number of posterior vertical bristles of 6 heads from flies raised at 18°C were quantified after imaging. (F-I) The same phenotypes from six flies raised at 25°C were quantified. Comparisons in each graph are made to the *dhipk* mutant (*dhipk* KO) result. “Control” flies are of the genotype +/+ ; *dhipk-Gal4/+.* Error bars indicate the mean with a 95% confidence interval. A one-way ANOVA was performed followed by Dunnett’s test to correct for multiple comparisons to the *dhipk* KO for each dataset. P-values for the statistical analyses performed correspond to the following symbols: ≥0.0332 (ns), <0.0332 (*), <0.0021(**), <0.0002(***), < 0.0001(****). Only female flies were assessed for this experiment.

**Supplemental Figure 3. Flies expressing *UAS-hHIPK1* in the eye-antennal disc do not develop aristae.** (A) Representative adult heads dissected from the corresponding genotypes. (B) Graphical representation of the *dpp-Gal4* domain in larval eye-antennal disc and adult head. Green indicates the *dpp-Gal4* domain, while other colors and patterns indicate corresponding regions between the larval and adult structures. Flies were raised at 29°C.

**Supplemental Figure 4. hHIPK1 induces Hox protein AbdB in wing, leg, and eye imaginal discs.**

Representative 3^rd^ instar imaginal (A) wing, (B) T_2_ leg, and (C) eye-antennal discs are shown for each of the corresponding crosses. Ectopic Abd-B staining caused by hHIPK1 is shown with arrows. Sex of the representative tissues are mixed unless otherwise noted by the male (♂) symbol. Crosses were performed at 29°C. Scale bars: 50µm.

**Supplemental Figure 5.** An 8-well Culture slide was modified as described in the materials and methods to facilitate preparation of multiple samples for high-resolution imaging on a single slide.

**Table S1. Hipks variably induce leg deformities and ectopic sex combs.**

Representative flies are shown in Fig 4A. Front legs are listed as T_1_, middle legs as T_2_, rear legs as T_3_. The penetrance of leg deformities for each genotype is separated by leg section, where Fe indicates the Femur, Ti indicates the Tibia, and Ta indicates the Tarsal segments, as indicated in Fig 4B. Both leg distortion and sex comb frequencies are listed for males only. Female legs are distorted, but frequencies are not listed here. No female legs from these genotypes display ectopic sex combs. Flies were raised at 29°C.
